# Development of an Affordable Silicone Phantom of The Kidney with 3D Printed Mould for Ultrasound Guided Percutaneous Nephrolithotomy Training in Indonesia

**DOI:** 10.12688/f1000research.156046.2

**Published:** 2025-07-23

**Authors:** Rananda Anggakara Hendarmo, Ponco Birowo, Nur Rasyid, Irfan Wahyudi, Gerhard Reinaldi Situmorang, Widi Atmoko, Mahesh Ramanlal Desai, Kevin Yonathan, Jonathan Pratama Swannjo

**Affiliations:** 1 Department of Urology, Universitas Indonesia – Cipto Mangunkusumo National General Hospital, Jakarta, DKI Jakarta, 10430, Indonesia; 2 Department of Urology, Muljibhai Patel Urological Hospital, Nadiad, Gujarat, India

**Keywords:** percutaneous nephrolithotripsy, model, surgery training, Op-simulation

## Abstract

**Objective:**

To develop an affordable kidney phantom for ultrasound guided percutaneous nephrolithotomy training model.

**Methods:**

Twenty one kidney models were manufactured and implemented as ultrasound guided percutaneous nephrolithotomy (PCNL) training models for urologists without any prior experience of independently performing PCNL in Indonesia. The unit cost of an alternative model was less than 30 USD (IDR 450,000). Twenty one junior with no prior experience of performing ultrasound guided PCNL participated in the research. The evaluation was done through anonymous responses to a questionnaire using the Likert scale.

**Result:**

The affordable PCNL model showed highly positive results among all participants (realistic anatomy 7.86/10, realistic visualization of calyx 8.19/10, realistic puncture 7.43/10, guidewire placement 8.19/10, realistic nephrostomy 7.57/10, stone visualization 7.76/10, feedback 7.52/10, post-training discussion 8.57/10). All of the participants recommended the affordable PCNL model phantom for ultrasound guided PCNL training in Indonesia.

**Conclusion:**

An affordable model utilizing 3D printed mould, silicone, and gelatine jelly is a feasible option for ultrasound guided PCNL training among urologists in developing countries. The utilization of 3D printing and silicone moulding will be beneficial in reducing the cost of surgery model while preserving the realistic tactile feedback.

## Introduction

Urolithiasis affects around 0.1–19.1% of the total population in Asia. ^1^ However, throughout the course of time, the frequency and incidence of the disease have shifted in a variety of nations or areas as a result of differences in socioeconomic standing as well as geographic locations. ^1^ Over the course of the past few decades, the frequency and incidence of urolithiasis have skyrocketed across the majority of the nations in Asia. ^1^ One of the most performed treatments for urolithiasis is percutaneous nephrolithotomy (PCNL). ^2^

For percutaneous nephrolithotripsy (PCNL) to be as effective as possible and to reduce any potential morbidity, it is imperative that the practitioner have sufficient training. Currently, training in PCNL is offered all over the globe via organized residency and fellowship programs. Individual theoretical and practical training courses are also offered to supplement this kind of education. However, there is some debate over the usefulness of the shorter training programs. ^3^

Surgical simulations are commonly performed in training for various urological procedures. ^4– 6^ However, commercially printed organ models and surgical simulators which use synthetic materials are typically lacking in anatomical details and often fabricated from materials with different sensations from the target organ. ^4^ Meanwhile, the use of animal tissues such as pig kidneys is usually cheaper but carries potential animal ethical tissues. ^2^

This study aims to develop a low-cost kidney transplantation surgery training model using both animal parts and synthetic materials.

## Methods

### Ethical considerations

The ethical approval to conduct this study was issued by the research ethic committee of the Faculty of Medicine, Universitas Indonesia with ethical clearance letter number KET-909/UN2.F1/ETIK/PPM.00.03/2022. Written informed consent was obtained from the urologists participating in this study. They were provided with a structured questionnaire to evaluate the training model’s realism, functionality, and overall effectiveness, using a Likert scale for scoring.

### Study design

This study is designed to develop and evaluate a low-cost PCNL training model using synthetic materials (silicone for kidney and gelatine jelly for the interstitial space) for both urologists and urology residents in developing countries such as Indonesia.

### Model development process

**3D printing of the kidney and calyceal system mould**

**Figure 1.**
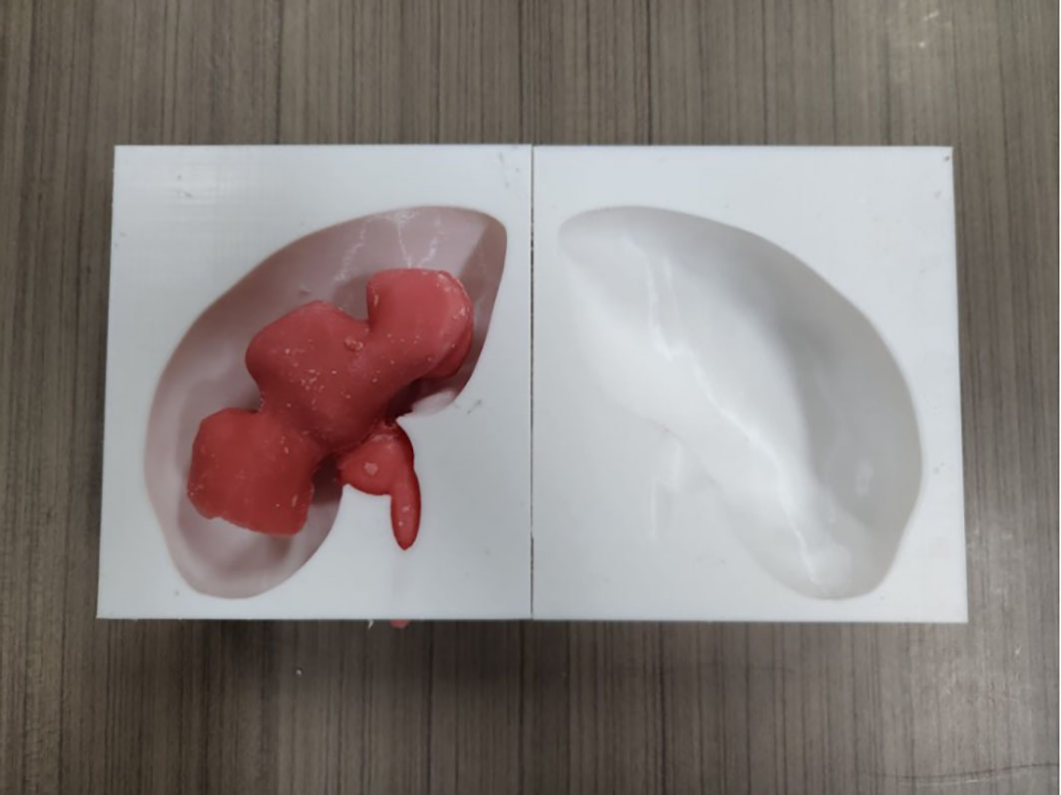
Initial mould of the kidney.

The kidney mould model was printed using a 3D-printer with Polylactid Acid (PLA) filament. The mould was separated into two interconnecting parts. Printing of a two-part mould took 25 hours and 288 grams of PLA filament. An adult kidney with hydronephrosis was scanned using computed tomography (CT) imaging. The DICOM file was deconstructed in InVesalius 3.0.0 (Centro de Tecnologia da Informação Renato Archer, Brazil, freely available on the web) and exported as STL (stereolithography) file. The file was then converted and stitched using a freely-available plug-in “Autodesk Mesh Mixer”. The STL file was printed out on a commercially available 3D printer (Ender-3 Pro, Creality 3D, China). Both the model of the kidney and calyceal system were printed as the initial mould ( Figure 1).

**Calyceal system moulding using wax**

**Figure 2.**
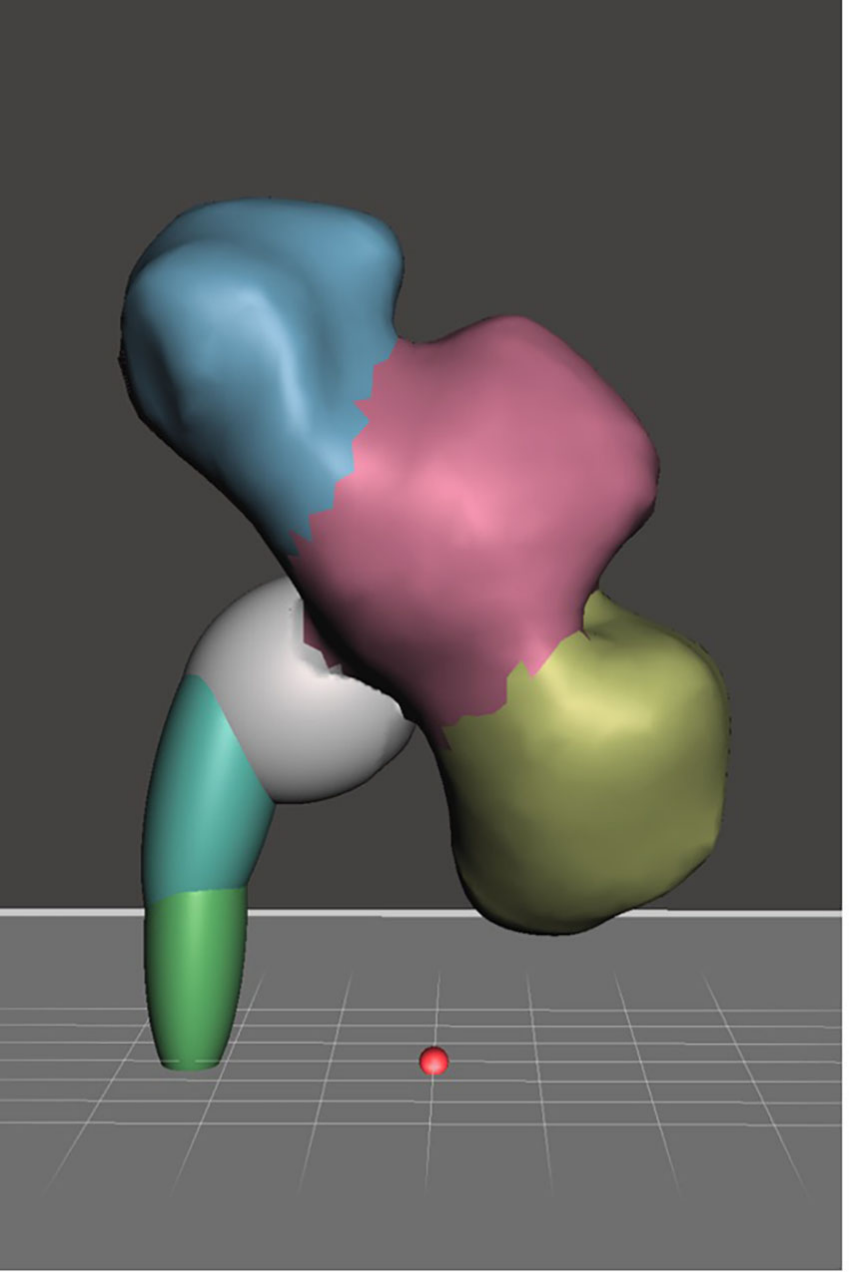
Calyceal system STL file.

Much like the kidney mould, the calyceal system was acquired from the same adult kidney with hydronephrosis scanned using CT imaging, the DICOM file was converted into STL file using InVesalius 3.0.0. STL refinement was done using Autodesk Mesh Mixer. The STL file of the calyceal system was printed with the same 3D printer. Printing of the calyceal system took 6 hours and 44 grams of PLA filament. The printed calyceal system was then casted in 400 mL of silicone RTV 52 with catalyst ratio of 1 to 100. In order to create a negative mould for further replication, wax casting of the calyceal system was done using 100 mL of commercially available paraffin wax ( Figure 2).

**Silicone kidney model assembly**

Following the development of both the 3D printed kidney mould and the wax calyceal system, the kidney was created using RTV52 silicone rubber that was poured into the mould with the wax calyceal system sandwiched between the two part mould. A total of 350 mL RTV52 silicone rubber was used to create one kidney with a catalyst ratio of 1 to 100. Following the curing time of the silicone, the silicone kidney was demoulded form the master mould and was boiled in water (80° C or 176° F) for 15 minutes to melt the wax calyceal system within the silicone kidney, leaving a hollow space inside the silicone kidney model.

**Figure 3.**
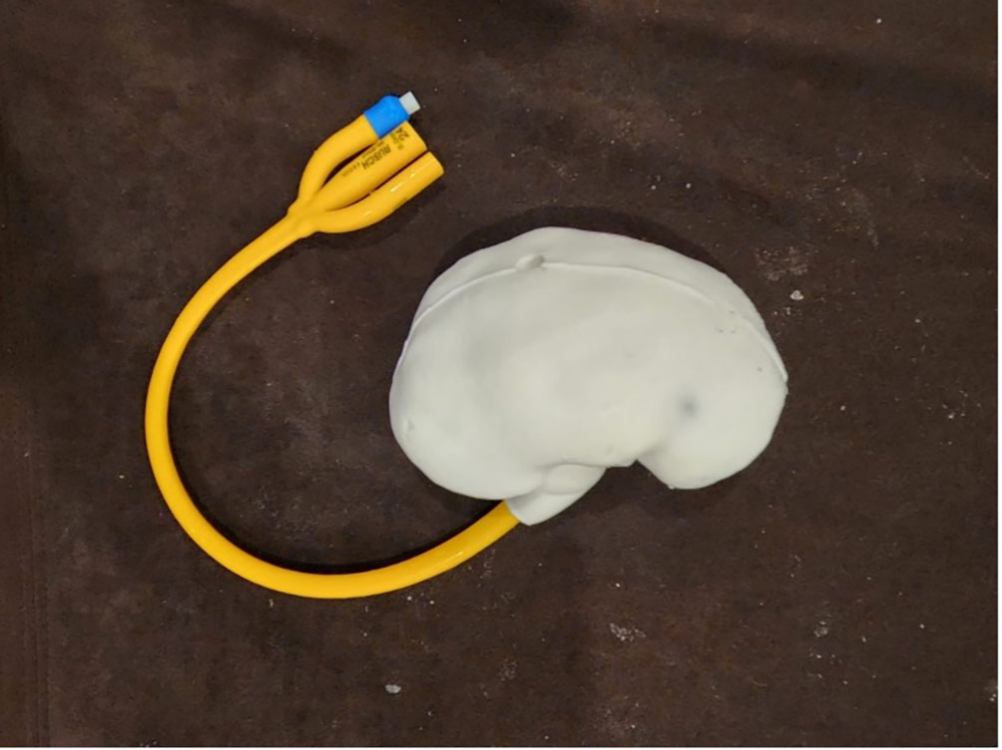
Assembled silicone kidney model.

Artificial stones made from commercially available camphor were inserted to facilitate nephrolithotripsy training. The kidney was sealed using a three-way foley catheter 24 Fr (Rusch®, Teleflex, USA) balloon to create a water sealed environment and simulate the closed urinary system. The foley catheter was clamped after adding tap water into the calyceal system ( Figure 3).

### Model assembly

**Figure 4.**
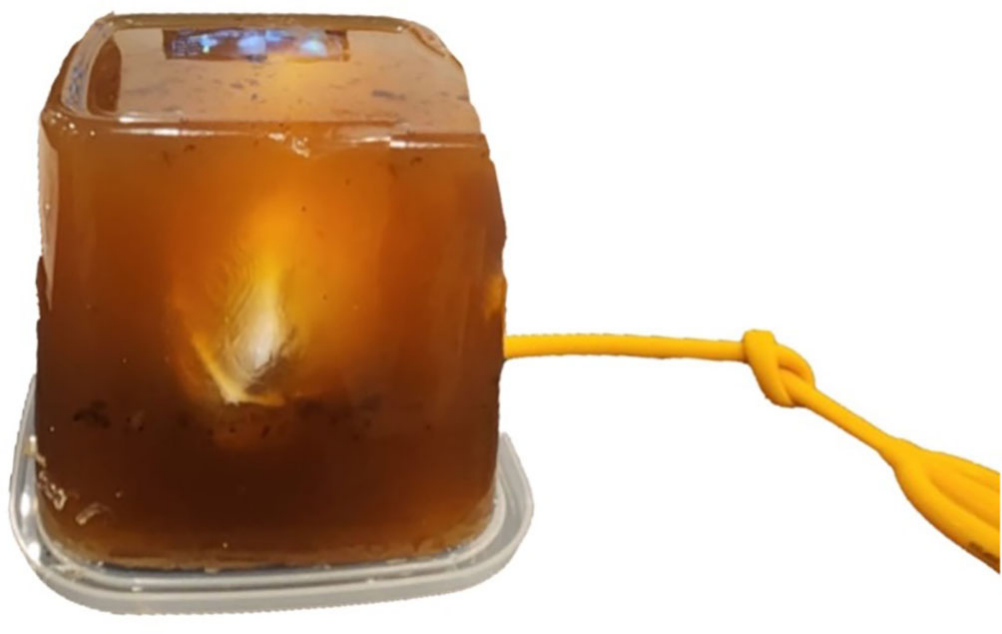
Final assembled model.

A 300 mL of gelatin medium was prepared from commercially available gelatin powder (culinary grade, no Bloom rating provided) mixed with water in a 1:9 ratio. The gelatine powder was added while boiling and stirring the water to avoid clumps. To ensure anatomical kidney angulation, the gelatin was poured in two stages. The first half was allowed to partially set before placing the kidney in its desired position, then the top half was poured afterward. The silicone kidney was submerged in a gelatine medium with a minimum depth of 7 cm to simulate the environment for PCNL procedure. The final model was then put into a chiller with a temperature of 4°C (40°F) for 6 hours to set the gelatine medium. The final model was then taken out from the chiller shortly before use ( Figure 4).

**Figure 5.**
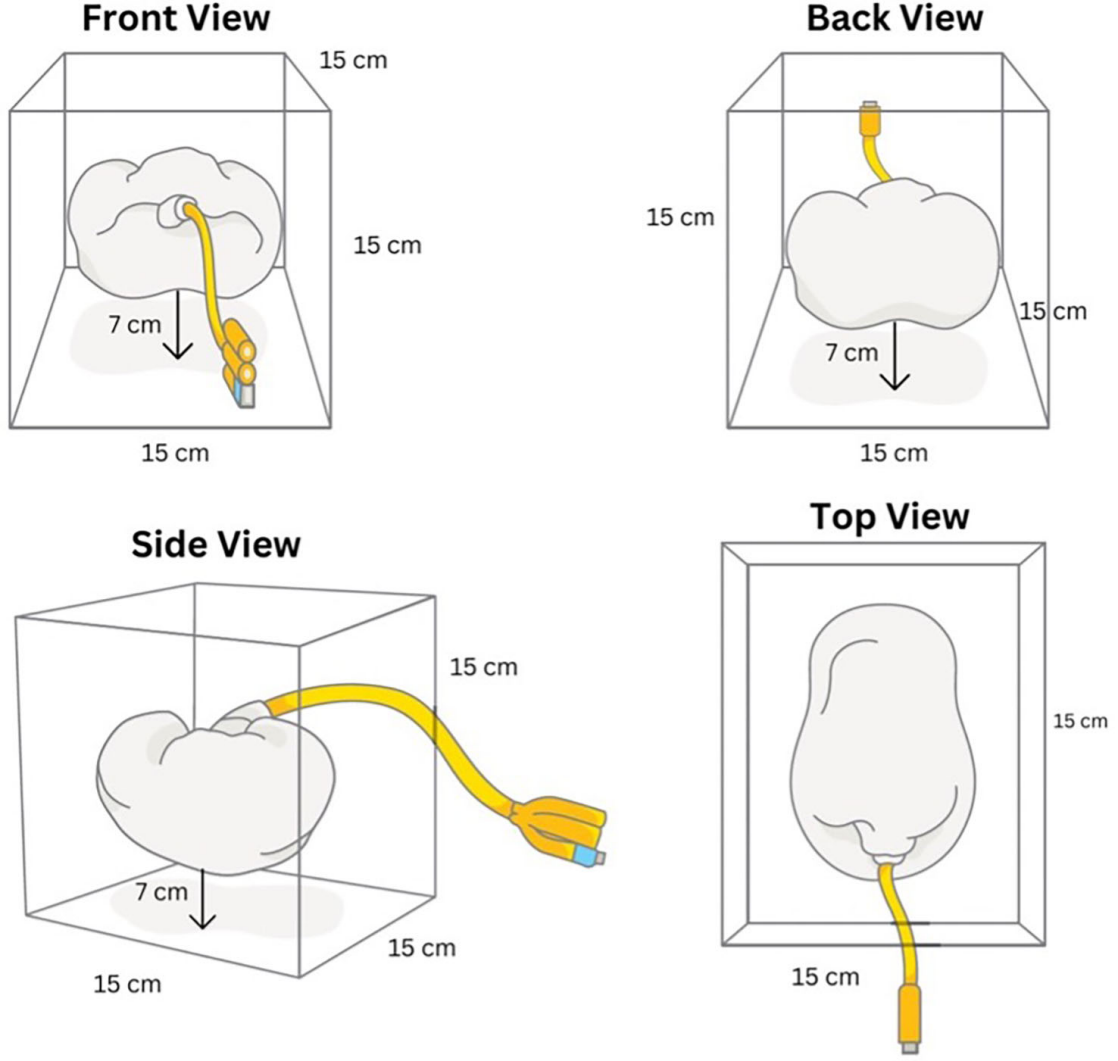
Model schematic.

### Ultrasound guided PCNL

The details of the surgical technique used in our training have been described in other publications. ^7^ Initially, an ultrasound convex ultrasound transducer with 3-5 megahertz (Mhz) frequency and needle-insertion bracket was used assess the calyceal system and identify route of insertion. Moreover, the distance between the outer part of the model (representing the skin) and the calyceal system was measured. A gravity bag of saline was hanged 60 cm above the model and connected to the foley catheter to simulate passive retrograde filling. The percutaneous renal access is established using a 17.5-gauge, echogenic, renal access needle.

Once the needle has reached the collecting system, removal of the needle stylet will facilitate visualization of normal saline (representing urine). The needle stylet is then withdrawn to allow the insertion of J-tip, superstiff, guidewire through the access needle. The guidewire was inserted until secured in place within the calyceal system.

**Figure 6.**
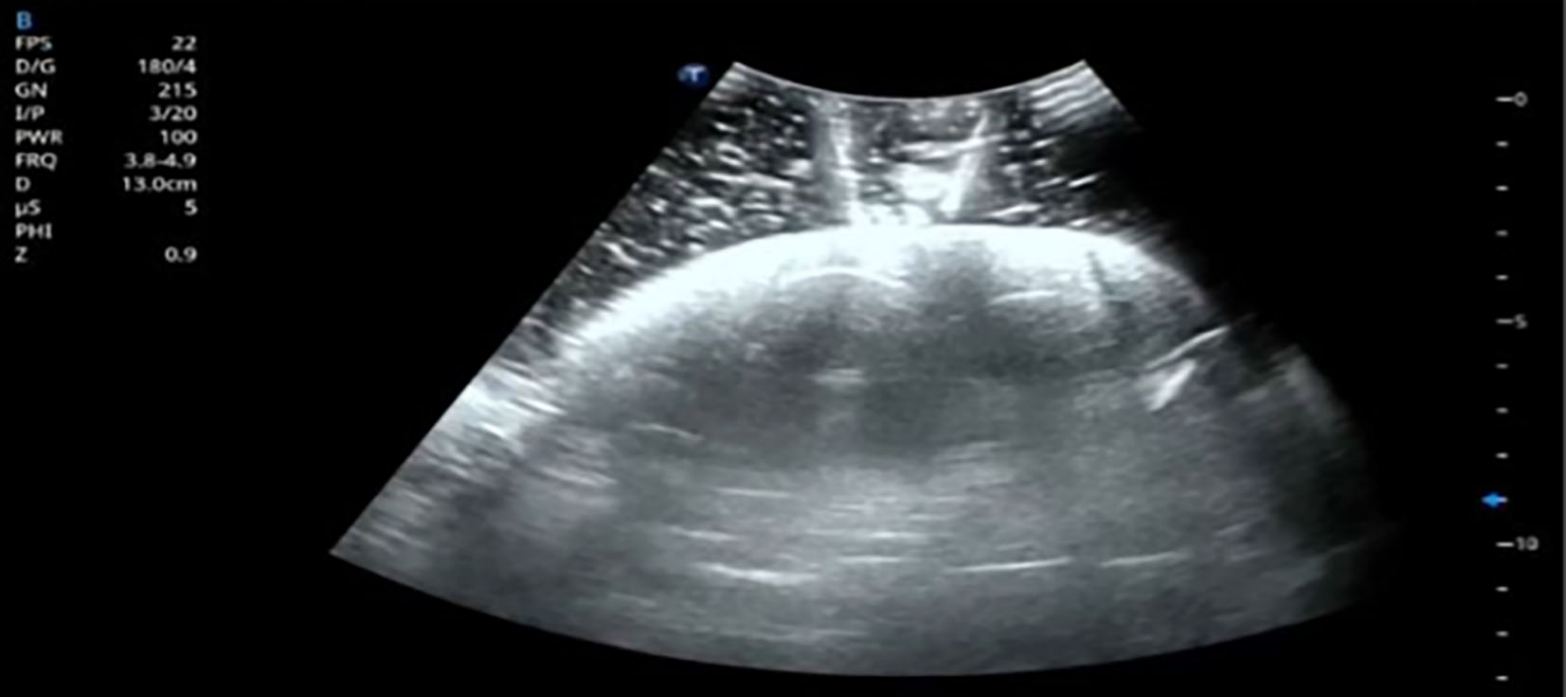
Ultrasound visualization of needle puncture through a silicone kidney model.

Following the guidewire insertion, a series of fascial dilator is passed over the guidewire until the measured distance to create a working tract. A percutaneous access sheath is then carefully advanced until the efflux of the normal saline is seen at the outer tip. Nephroscopy for stone removal and nephrolithotripsy are then done as per usual standard fluoroscopy-guided PCNL. An ultrasound image showing the needle entering the simulated calyceal system has been added ( Figure 6) to demonstrate the real-time visualization during ultrasound-guided access. Although silicone provides adequate tactile sensation, one of its drawbacks is that ultrasound imaging cannot visualize the calyces effectively due to its acoustic properties.

### Evaluation of the model

The evaluation of our model was done through an anonymous response to a questionnaire developed by Ali et al (2020). ^8^ The questionnaire consisted of 8 primary questions with a Likert scale of 1 (strongly disagree) to 10 (strongly agree). A higher score represented a better perception from the respondent. Moreover, an additional comment and suggestion section was placed following the questionnaire. The complete contents of the questionnaire can be found in Table 1.

**Table 1.** PCNL training model likert scale questionnaire.

Question	Likert scale (1-10)
	Median (Q1-Q3)
Q1: How do you rate the realism of kidney anatomy evaluation using ultrasound?	
Q2: How do you rate the realism and visualization of a calyx for puncture?	
Q3: How do you rate the US guided puncture of the pelvicalyceal system?	
Q4: How do you rate the realism of a guidewire placement?	
Q5: How do you rate the realism of nephrostomy tube placement?	
Q6: How can you describe the stone shape and its location?	
Q7: How do you rate the realism of tissue model feedback?	
Q8: How do your rate the benefit of post-training errors discussion?	

Notes: Likert score interpretation: 1 strongly disagree, 2 disagree, 3 neutral, 4 agree, 5 strongly agree.

## Results

During the study, a total of 21 urologists with no experience of performing US-guided PCNL in Indonesia participated in the training and anonymously gave the evaluation of our model. The questionnaire used can be found in Table 1, whereas the result of the perception of the model could be found in Table 2.

**Table 2.** Urologists’ perception toward the PCNL training model score.

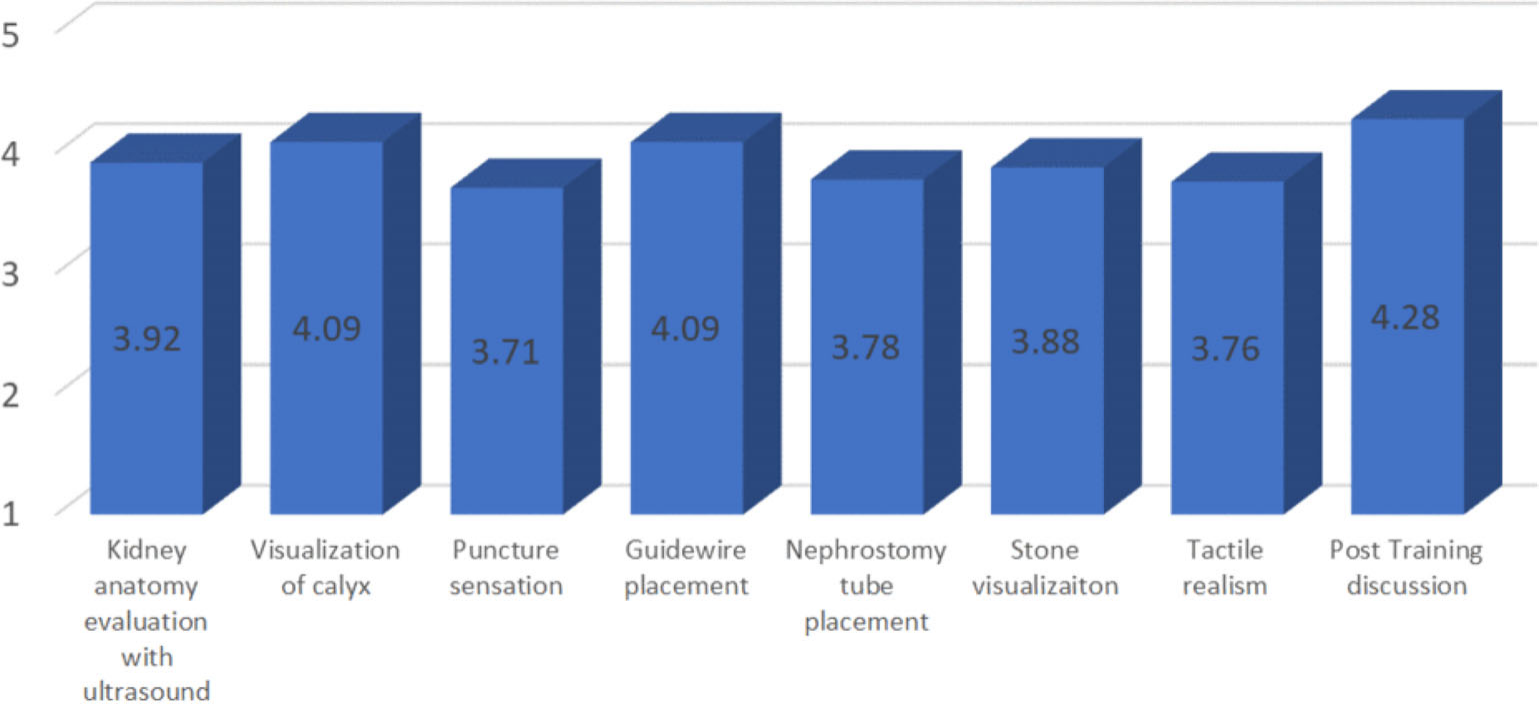

Based on the evaluation, there were highly positive results among participants (average score of kidney anatomy evaluation using ultrasound 3.92/5, visualization of calyx 4.08/5, puncture sensation 3.71/5, guidewire placement 4.09/5, nephrostomy tube placement 3.78/5, Stone visualization 3.88/5, Tactile realism 3.76/5 and Post training discussion 4.28/5. The question with the lowest average score was about puncture sensation. However, there was no additional comment or suggestion regarding the issue.

## Discussion

In this study, we have developed an affordable non-biological model using with similar characteristics to the kidney for percutaneous nephrolithotomy (PCNL) training. Based on the post-training evaluation of our model, a high satisfactory rate was obtained from every participant in our study.

The results of our study was in line with other PCNL training model created in other studies. ^8, 9^ Although traditionally PCNL training consists of dry lab training using commercially available bench model and wet lab training on real patients, the cost of traditional model and the safety of the patients remain as an issue. Moreover, training using animal models such as porcine kidney may not fully represent the calyceal system in human, while training using virtual reality (VR) simulation model is currently very limited in developing countries such as Indonesia. ^10^

For surgical teaching systems, there are a variety of possibilities, including synthetic models, virtual reality simulations, animal parts, and complete animals. ^2^ Synthetic models are frequently selected for training in several teaching hospitals and educational institutions due to their nature as the comparatively affordable, simply available, and repeatedly reusable model. When executing the procedure, it is, however, similarly deficient in actual tactile input. On the other hand, whole animals and reality simulations are expensive and challenging to obtain. ^2^ To offer a comparable experience while still keeping the cost of the model low, we thus sought to use an affordable synthetic model with realistic consistency.

The hands-on training using animal model and live surgery is considered useful in changing the practices and techniques in PCNL, even for attending surgeons. ^3^ We hypothesized that the hands-on training using non-biological model would also provide similar experience and changes to the current practice of the urologists in developing countries.

One limitation of this study is the lack of expert urologist validation prior to involving novices. Due to logistical constraints, only urologists without prior PCNL experience participated. Future studies are planned to include validation by experienced PCNL practitioners to enhance model reliability.

Currently, the fluoroscopy guidance for PCNL is the most commonly used imaging modality for the surgery, starting from obtaining renal access to performing stone extraction. However, radiation exposure is an important issue for patients with urolithiasis as it tends to form repeatedly during a lifetime. ^11^ Thus, the rising use of ultrasound-guided PCNL is deemed as an alternative with fewer risk of radiation exposure. ^12^ There are only limited options of training model for ultrasound-guided PCNL. Therefore, we hope that the development of our training model to be beneficial in improving the learning curve of ultrasound-guided PCNL.

The limitation of this study is the lack of comparison between the developed model and a similar model. Moreover, there is no objective measurement of the models’ characteristics, such as consistency, plasticity, and tensile strength.

## Conclusion

An affordable model utilizing 3D printed mould, silicone, and gelatine jelly is a feasible option for ultrasound guided PCNL training among urologists in developing countries. The utilization of 3D printing and silicone moulding will be beneficial in reducing the cost of surgery model while preserving the realistic tactile feedback.

## Ethics and consent

The ethical approval to conduct this study was issued by the Research Ethics Committee of the Faculty of Medicine, Universitas Indonesia, with ethical clearance letter number KET-909/UN2.F1/ETIK/PPM.00.03/2022, dated 22 September 2022. Written informed consent was obtained from the subjects participating in the study. This study complies with the Declaration of Helsinki and was performed according to ethics committee approval.

## Registry and Registration Number of the Study

Not applicable.

## Authors’ contributions

**RAH:** Conceptualization, Data curation, Formal analysis, Investigation, Resources, Software, Methodology.

**PB:** Conceptualization, Data curation, Formal analysis, Investigation, Project administration, Resources, Software, Validation, Methodology, Writing, Supervision, Funding acquisition.

**NR:** Conceptualization, Formal analysis, Investigation, Project administration, Software, Validation, Methodology, Writing, Supervision, Funding acquisition.

**IW:** Conceptualization, Data curation, Formal analysis, Investigation, Project administration, Writing, Supervision, Funding acquisition.

**GRS:** Conceptualization, Data curation, Formal analysis, Investigation, Project administration, Writing, Supervision, Funding acquisition.

**WA:** Formal analysis, Investigation, Software, Validation, Methodology, Writing, Supervision, Funding acquisition.

**MRD:** Conceptualization, Data curation, Formal analysis, Investigation, Writing, Supervision

**KY:** Conceptualization, Data curation, Formal analysis, Investigation, Validation, Methodology, Writing.

**JPS:** Conceptualization, Investigation, Project administration, Resources, Validation, Writing.

**All authors** has approved the final version of the manuscript.

## Ethical clearance

Ethical clearance for this study was given by The Research Ethics Committee, Faculty of Medicine, Universitas Indonesia with ethical clearance number KET-909/UN2.F1/ETIK/PPM.00.02/2022. This study conformed with Declaration of Helsinki and its subsequent amendments.

## Data Availability

The data associated with this study is protected under HAKI (Hak Atas Kekayaan Intelektual) in Indonesia, which restricts the sharing of detailed data outside of the research team. The Institutional Review Board has mandated that access to the data can only be granted under specific conditions that comply with national intellectual property regulations. Any requests for access to the data must be formally submitted and will be reviewed by the appropriate legal and ethical authorities to ensure compliance with these regulations. The data generated during this study has been deidentified in accordance with the Safe Harbour method to ensure participant confidentiality. All authors involved in the creation of the dataset have been credited. Development of an Affordable Silicone Phantom of The Kidney with 3D Printed Mould for Ultrasound Guided Percutaneous Nephrolithotomy Training in Indonesia, https://doi.org/10.17605/OSF.IO/EH4YB. ^13^ Data are available under the terms of the Creative Commons Zero “No rights reserved” data waiver (CC0 1.0 Public domain dedication). This study follows the STROBE (Strengthening the Reporting of Observational Studies in Epidemiology) checklist to ensure clear and detailed reporting of the research design, methods, and findings. Comprehensive information on participant recruitment, model development, and evaluation has been included to allow reproducibility of the work, https://doi.org/10.17605/OSF.IO/EH4YB. ^13^ Data are available under the terms of the Creative Commons Zero “No rights reserved” data waiver (CC0 1.0 Public domain dedication).
