# Supplementary material for: Development of an Affordable Silicone Phantom of The Kidney with 3D Printed Mould for Ultrasound Guided Percutaneous Nephrolithotomy Training in Indonesia
Source: F1000Res. 2025 Jul 23;14:237. Originally published 2025 Feb 26. [Version 2] doi: 10.12688/f1000research.156046.2 (PMC12381629; doi:10.12688/f1000research.156046.2)
Supplement: Peer review history

# Reviewer report: Reviewer response for version 2

**DOI:** 10.5256/f1000research.184097.r400177

**Published:** 2025-08-23

**Authors:** Enrique Pulido-Contreras

**Affiliations:** 1 Unidad Médica de Alta Especialidad del Bajío, Instituto Mexicano del Seguro Social (IMSS), León, Mexico

I have no further comments regarding this manuscript. 

 Congratulations on your work.

Is the work clearly and accurately presented and does it cite the current literature?

Yes

If applicable, is the statistical analysis and its interpretation appropriate?

I cannot comment. A qualified statistician is required.

Are all the source data underlying the results available to ensure full reproducibility?

Yes

Is the study design appropriate and is the work technically sound?

Yes

Are the conclusions drawn adequately supported by the results?

Yes

Are sufficient details of methods and analysis provided to allow replication by others?

Yes

Reviewer Expertise:

Ultrasound guided Percutaneous Nephrolithotomy and Endoscopic Enucleation of the prostate with Bipolar energy.

I confirm that I have read this submission and believe that I have an appropriate level of expertise to confirm that it is of an acceptable scientific standard.

# Reviewer report: Reviewer response for version 1

**DOI:** 10.5256/f1000research.171303.r377617

**Published:** 2025-06-03

**Authors:** Ben H. Chew

**Affiliations:** 1 University of British Columbia, Vancouver, Canada

the authors describe a novel method that is easy to make, reproducible and is a high fidelity model to represent ultrasound guidance of kidney puncture. it is easy to produce, cost effective and the surgeons who tested it found it to be highly representative. 

 The manuscript is well written and presented with excellent figures and photographs describing how to produce this.

Is the work clearly and accurately presented and does it cite the current literature?

Yes

If applicable, is the statistical analysis and its interpretation appropriate?

Yes

Are all the source data underlying the results available to ensure full reproducibility?

Yes

Is the study design appropriate and is the work technically sound?

Yes

Are the conclusions drawn adequately supported by the results?

Yes

Are sufficient details of methods and analysis provided to allow replication by others?

Yes

Reviewer Expertise:

kidney stones

I confirm that I have read this submission and believe that I have an appropriate level of expertise to confirm that it is of an acceptable scientific standard.

# Reviewer report: Reviewer response for version 1

**DOI:** 10.5256/f1000research.171303.r379885

**Published:** 2025-05-14

**Authors:** Enrique Pulido-Contreras

**Affiliations:** 1 Unidad Médica de Alta Especialidad del Bajío, Instituto Mexicano del Seguro Social (IMSS), León, Mexico

Thank you for the opportunity to review your manuscript. An interesting work on a low-cost phantom model useful to simulate percutaneous access under ultrasound guidance. The search for models as real as possible for the urologist without experience in ultrasound-guided access or the urology resident to learn the basics of puncture without exposing the patient to risks is of utmost importance and should be implemented in all centers. Congratulations on your work.

 May I make some observations to improve the quality of the work:

 1.             In the preparation of the model, I think it is important to specify what type of gelatin was used. There is a classification of the hardness of gelatin known as Blooms, so it would be good to specify the type of gelatin used so that the hardness would be sufficient for the model to be used for multiple punctures. It would also be good to add if they used Psyllium Plantago so that the gelatin would have a texture similar to fat tissue in the ultrasound. Also, due to the density of the gelatin, less dense materials tend to ascend to the surface, did you use a mechanism to hold it in place and give the kidney a natural angulation?

 2. Real Images of the model's ultrasound should be added or placed where the needle can be visualized entering the simulated collecting system. This will allow us to see how the model is seen in the ultrasound machine.

 3. It has not been done, and perhaps it would be a complex request to achieve. Still, in the validation of the phantom model, the ideal would have been that first, a group of urologists experts in percutaneous ultrasound guided nephrolithotomy would validate their experience and opinions of the model, the process of including the opinion of experts is crucial to ensure the accuracy and reliability of the model (refer to 1 ) and then compare it with the group of urologists without experience, if possible to make this comparison would increase the quality of their work.

 In general, I think that your model is great and allows to practice the puncture and the different dilatation techniques completely guided by the Ultrasound, gaining experience to be able to perform radiation free PCNL.

Is the work clearly and accurately presented and does it cite the current literature?

Yes

If applicable, is the statistical analysis and its interpretation appropriate?

I cannot comment. A qualified statistician is required.

Are all the source data underlying the results available to ensure full reproducibility?

Yes

Is the study design appropriate and is the work technically sound?

Yes

Are the conclusions drawn adequately supported by the results?

Yes

Are sufficient details of methods and analysis provided to allow replication by others?

Yes

Reviewer Expertise:

Ultrasound guided Percutaneous Nephrolithotomy and Endoscopic Enucleation of the prostate with Bipolar energy.

I confirm that I have read this submission and believe that I have an appropriate level of expertise to confirm that it is of an acceptable scientific standard, however I have significant reservations, as outlined above.

## References

[1] : Fundamental principles of validation, and reliability: rigorous science for the assessment of surgical education and training. *Surg Endosc* .2003;17(10) : 10.1007/s00464-003-0035-4 1525-9 10.1007/s00464-003-0035-4 14502403
